# Impact of COVID-19 on the mental health of hospital staff

**DOI:** 10.1192/j.eurpsy.2023.1713

**Published:** 2023-07-19

**Authors:** S. Chemingui, I. Yousfi, N. Mechergui, M. Mersni, S. Ernez, D. Brahim, H. Ben Said, G. Bahri, I. Youssef, N. Ladhari

**Affiliations:** 1 Department of Occupational Medicine, Charles Nicolle Hospital of Tunis, Tunis, Tunisia

## Abstract

**Introduction:**

The COVID-19 pandemic had a significant psychological and physical impact throughout the world. Indeed, the rapid increase in the number of cases of infection created stressful situations and an anxiety-inducing climate that significantly affected the mental health of the world’s population, particularly that of healthcare workers (HCWs) who were massively mobilized to deal with the crisis.

**Objectives:**

To assess the frequency of anxiety-depressive disorders in HCWs who have contracted the SARS-Cov2 virus.

**Methods:**

Cross-sectional descriptive study interested the HCWs of the Charles Nicolle Hospital of Tunis having had COVID-19 during the period from September 1, 2020, to December 31, 2020. The psychological impact was studied through the HAD questionnaire (anxiety and depression assessment scale), administered to hospital workers at the time of the medical visit to return to work.

**Results:**

The study population consisted of 531 Hcws. The mean age was 40 years with extremes ranging from 24 to 63 years. A female predominance of 76.6% was noted. The average professional seniority was 10 years [one year-37 years]. Nurses were the main professional category (32.4%). The study population belonged mainly to the departments of gynecology (8.3%), general surgery (7.2%), internal medicine (6.4%), and emergency (5.5%). A pathological history was found in 89.6% of cases, 7.2% of which were psychiatric. Anxiety (total score >10) was noted in 36.5% of patients. On the other hand, a certain depression (total score “depression” >10) was found in 33.3% of HCWs.

**Conclusions:**

The COVID-19 pandemic induced a significant psychological impact on the HCWs placed in the first line in the management of this health crisis. As a result, long-term psychological follow-up of healthcare workers is essential in order to preserve health at work in care settings.

**Disclosure of Interest:**

None Declared

